# *Borrelia afzelii* Hepatitis in Patient Treated with Venetoclax and Obinutuzumab, Switzerland

**DOI:** 10.3201/eid3111.250584

**Published:** 2025-11

**Authors:** Gioele Capoferri, Raphael Battegay, Baptiste Hamelin, Peter M. Keller, Kirsten D. Mertz, Maja Weisser

**Affiliations:** University Hospital Basel, Basel, Switzerland

**Keywords:** *Borrelia afzelii*, vector-borne infections, bacteria, Lyme borreliosis, hepatitis, immunosuppression, molecular diagnostics

## Abstract

We report *Borrelia afzelii* hepatitis in an immunosuppressed patient in Switzerland receiving anti-CD20 therapy and venetoclax. Diagnosis was made by metagenomic sequencing and PCR. This case underscores the need to consider Lyme borreliosis in unexplained hepatitis cases and highlights the value of molecular diagnostics in immunosuppressed patients when serologic test results are negative.

Lyme borreliosis (LB) presents a wide range of clinical manifestations across its stages. Early localized infection (stage 1) typically manifests as erythema migrans, whereas later stages involve systemic complications (*1*,*2*). In North America, *Borrelia burgdorferi* sensu stricto is the predominant causative agent (*3*). Possible manifestations of early dissemination are multiple erythema migrans, arthritis, or acute neuroborreliosis (*1*,*2*). In Europe, *B. afzelii* and *B. garinii* are more common causes (*3*); *B. afzelii* is the most frequent cause of erythema migrans, lymphocytoma, and acrodermatitis chronica atrophicans, and *B.* *garinii* primarily causes neuroborreliosis (*1*,*2*). Although mild hepatopathy occurs in up to 27% of LB cases in the United States (*4*) and in 14%–15% of cases in Europe (*5*), hepatic infection by *Borrelia* spp. is rare (*6*). We report a case of hepatic infection caused by *B. afzelii* in a patient in Switzerland with chronic lymphocytic leukemia (CLL) receiving venetoclax and obinutuzumab. Written informed consent for participation in this case report was obtained from the patient by the authors.

## The Case

**Figure 1:**
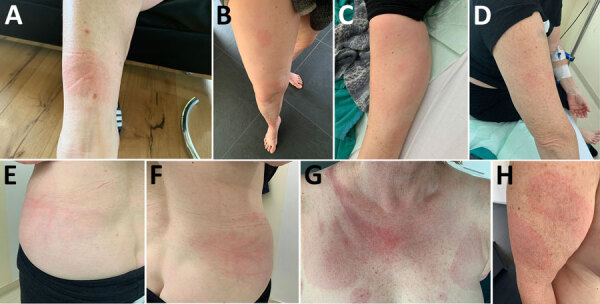
Evolution of cutaneous findings for reported case of early disseminated Lyme borreliosis with multiple erythema migrans and hepatitis in 62-year-old immunosuppressed patient treated with venetoclax and obinutuzumab, Switzerland. A) Oval erythematous rash on the anterior aspect of the right ankle, first lesion to appear (February 20, 2024). B) Right leg with 2 new additional circular/oval patches located on the anterior thigh and anterior knee (March 3, 2024). C–F) Worsening of the exanthemas, with expanding red rash and central clearing (targetoid appearance) on the left leg (C) and onset of red macules on the right arm (D) and the left (E) and right (F) posterolateral trunk (April 9, 2024). G–H) Macules on the décolleté (G) and evolution of cutaneous findings on the right arm, including the appearance of an additional macule (H) (April 27, 2024).

**Figure 2:**
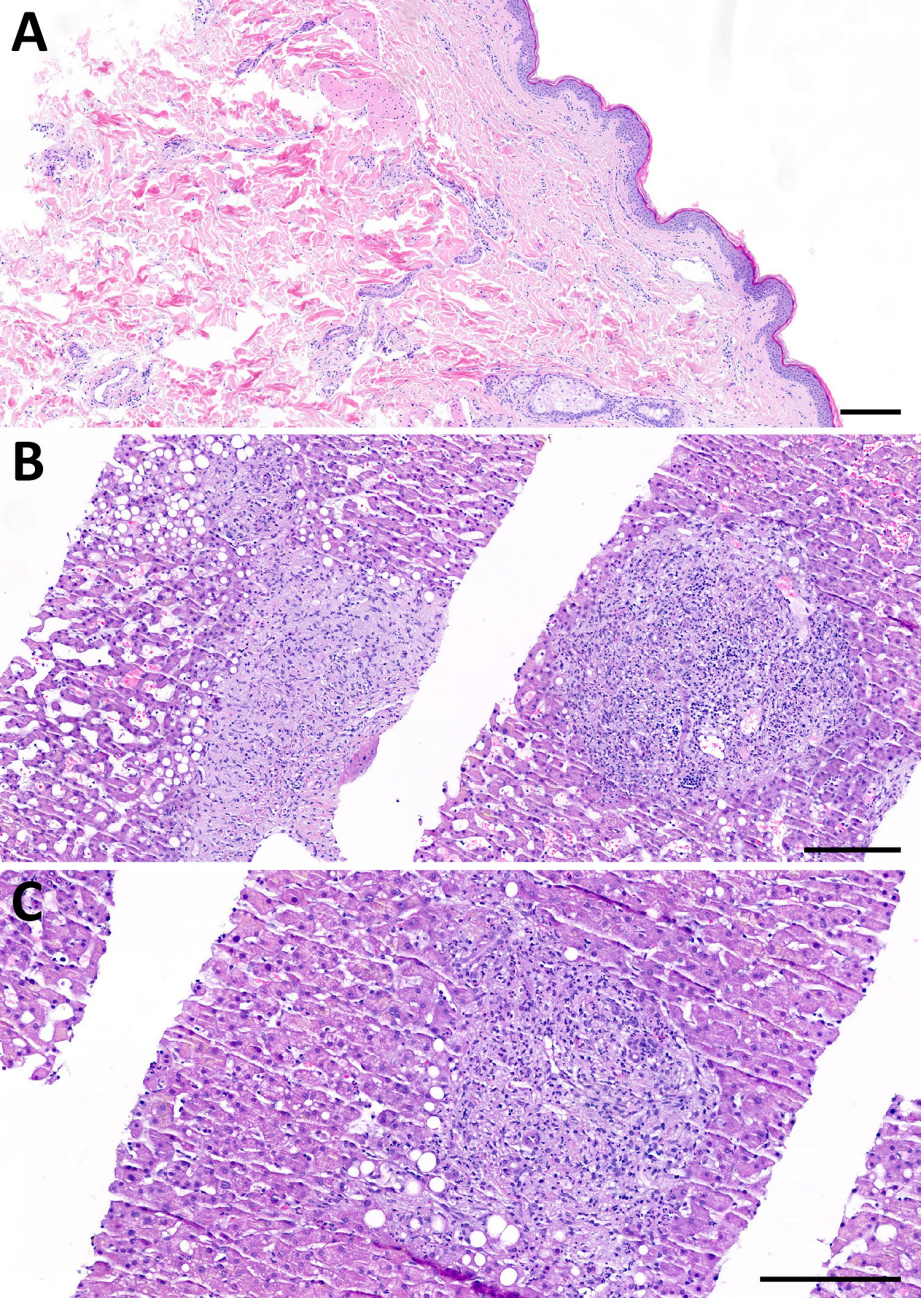
Histopathology of skin and liver for reported case of early disseminated Lyme borreliosis with multiple erythema migrans and hepatitis in a 62-year-old immunosuppressed patient treated with venetoclax and obinutuzumab, Switzerland. A) Skin biopsy from an erythema chronicum migrans lesion on the gluteal region, stained with hematoxylin and eosin. The histology reveals a mild superficial and deep perivascular dermatitis characterized by a sparse, predominantly lymphocytic inflammatory infiltrate, without evidence of plasma cells. B–C) Liver biopsy stained with hematoxylin and eosin showing features of chronic cholestatic hepatitis with superimposed acute cholangitis, while maintaining an overall preserved lobular architecture. Key histological findings include marked sinusoidal dilatation (peliosis); expanded, markedly edematous portal tracts without fibrosis; and prominent ductular proliferates with intraepithelial and intraluminal neutrophilic infiltrates consistent with neutrophilic cholangitis. Neutrophilic infiltrates were also observed within the sinusoids. Additional features included portal ceroid-laden macrophages and microvesicular steatosis. Scale bars indicate 200 µm.

A 62-year-old white woman with CLL diagnosed in 2016 was managed with watchful waiting until late 2023, when biopsy-confirmed leukemia cutis developed on her right shoulder. In March 2024, she began a chemotherapy regimen of obinutuzumab, a novel anti-CD20 monoclonal antibody, and venetoclax. One week later, she reported nonpruritic erythematous rashes on her legs; the first appeared on the right ankle, and additional rashes spread to the right and left leg. Clinically, multiple circular erythematous exanthemas with a maximum diameter of 10 cm were present on both legs (Figure 1). After 2 weeks, the rash on the left leg had further expanded, exhibiting discrete central clearing (Figure 1). In addition to that progression, similar new exanthemas appeared on the trunk and both arms (Figure 1). A skin biopsy showed a mild superficial and deep lymphocytic perivascular dermatitis (Figure 2, panel A). Initial test results for *Borrelia* IgM were negative; borderline IgG elevation was noted (10.78 AU/mL [reference <10 AU/mL]) (Diasorin, https://int.diasorin.com). Results of immunoblot (Virotech, http://www.virotechdiagnostics.com) were positive only for variable major protein-like sequence, expressed. Topical steroids were initiated for suspected morphea.

One month later, the exanthemas showed slight improvement, except for those on the right arm and upper chest (Figure 1). At that time, the patient began experiencing persistent fever of temperatures exceeding 38.5°C and elevated C-reactive protein (84 mg/L [reference <10 mg/L]); leukocyte and transaminase levels were within reference ranges, but cholestatic parameters were mildly elevated (gamma-glutamyl transferase 55 U/L [reference 6–40 U/L]; alkaline phosphatase 317 U/L [reference 35–105 U/L]). Results of a whole-body computed tomography scan were unremarkable. A drug fever was suspected; venetoclax and obinutuzumab were discontinued in June 2024. Three weeks later, the patient was hospitalized for persistent fever, worsening cholestatic parameters (gamma-glutamyl transferase189 U/L, alkaline phosphatase 804 U/L), new hepatitis (aspartate transferase 140 U/L [reference 11–34 U/L], alanine transaminase 80 U/L [reference 8–41 U/L]), and elevated C-reactive protein (120 mg/L). Results of serologic testing and PCR were negative for hepatitis B, C, and E; Epstein-Barr virus; cytomegalovirus; herpes simplex virus; adenovirus; HIV; *Bartonella henselae*; *Coxiella burnetii*; *Brucella* spp.; *Toxoplasma gondii*; *Schistosoma* spp.; *Leishmania* spp.; and dimorphic fungi. Results of blood cultures, including those for mycobacteria, were negative, as was follow-up serologic testing for *Borrelia*. Liver ultrasound revealed discrete hepatomegaly with mildly mottled parenchyma; Fibroscan (Echosens, https://www.echosens.com/en-us) showed a slightly elevated stiffness (7.6 kPa [reference 2–7 kPa]). Positron emission tomography–computed tomography showed global hepatic hypermetabolism. Liver biopsy showed chronic cholestatic hepatitis with acute cholangitis but preserved lobular architecture (Figure 2, panels B, C). Immunohistochemistry results for cytomegalovirus and Epstein-Barr virus were negative; unfortunately, no sample for microbiology was taken. To further investigate the origin of hepatitis, metagenomic next-generation sequencing (mNGS) performed on the liver biopsy identified *B. afzelii*, which was confirmed by nested PCR (Appendix). Retrospective PCR analysis of the skin biopsy was also positive for *B. afzelii*, establishing the diagnosis of early disseminated LB with multiple erythema migrans and hepatitis.

**Figure 3:**
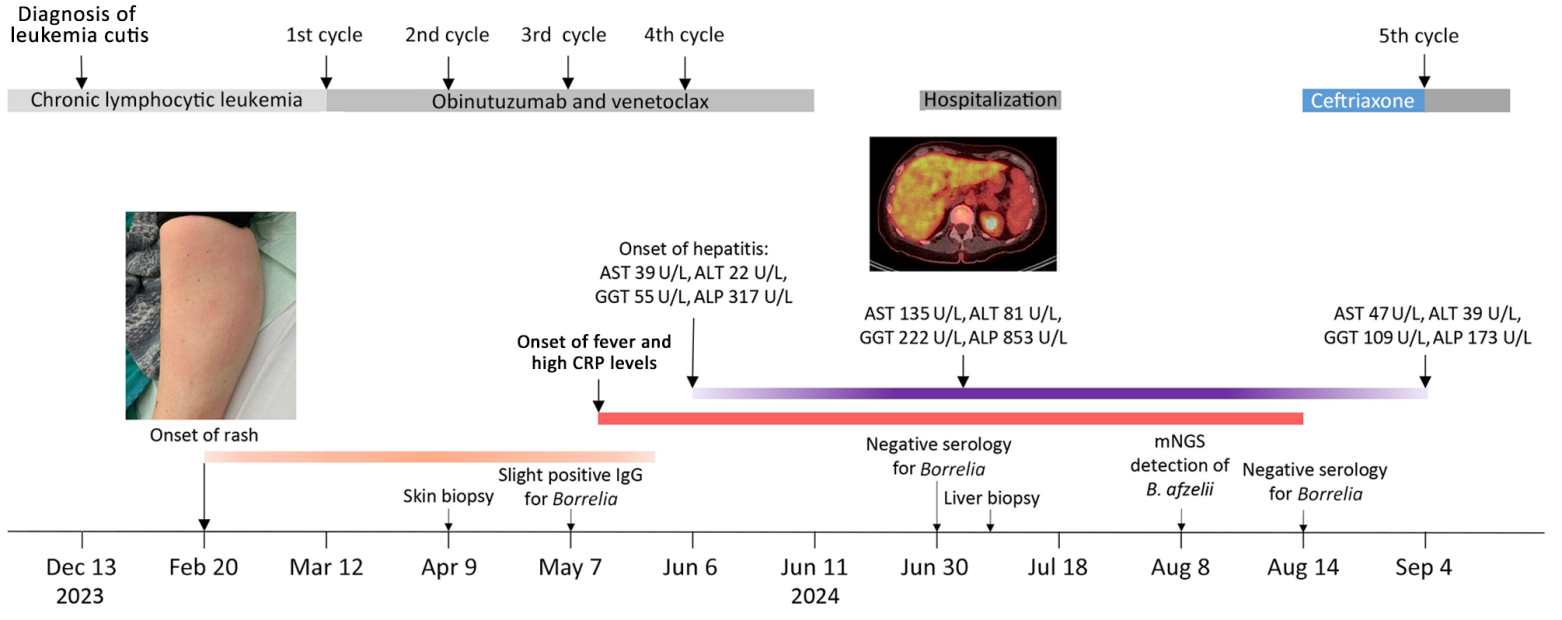
Timeline for reported case of early disseminated Lyme borreliosis with multiple erythema migrans and hepatitis in a 62-year-old immunosuppressed patient treated with venetoclax and obinutuzumab, Switzerland. ALP, alkaline phosphatase; ALT, alanine transaminase; AST, aspartate transferase; CRP, C-reactive protein; GGT, gamma-glutamyl transferase; mNGS, metagenomic next-generation sequencing.

The patient was treated with ceftriaxone (2 g/d for 3 wks) and fully recovered (Figure 3). Patient history revealed tick bites in the year before the onset of symptoms. The patient, who resides in a small village in a valley in Switzerland, had been treated for erythema migrans ≈20–30 years previously.

## Conclusions

We documented a case of *B. afzelii* hepatitis in an immunosuppressed patient receiving anti-CD20 therapy and venetoclax. The case underscores the diagnostic challenge of LB in severely immunocompromised persons, in whom serologic testing might be unreliable and mNGS offers a valuable diagnostic tool.

We found only 6 cases of histologically confirmed *Borrelia*-associated hepatitis in the literature; of those, 2 cases were PCR-confirmed (*B. garinii* and *B. burgdorferi* s.s.) (*7*–*12*) (Appendix Table). Histology typically showed sinusoidal and portal inflammation, Kupffer cell hyperplasia, granulomatous hepatitis, or a combination of those; spirochetes were visualized in 2 cases. Clinically, most hepatitis manifested in fever and nonspecific symptoms. Abdominal pain was reported in 1 case and erythema migrans in another case.

The pathogenesis of hepatic injury in LB likely involves direct hepatic infiltration by spirochetes and immune-mediated damage (*4*). An in vitro study suggests that vascular adhesion and emigration might represent key strategies used by *B. burgdorferi* sensu lato to evade the intravascular innate immune response, enabling persistence in organs such as the liver (*13*). Subsequent inflammatory infiltration and Kupffer cell activation contribute to hepatic injury (*14*). In our case, although Warthin-Starry staining did not reveal spirochetes, its limited sensitivity and strong background staining do not exclude presence of spirochetes in the liver tissue. The pronounced granulomatous inflammation, together with elevated liver enzymes and hepatomegaly, strongly suggests local hepatic involvement rather than a passive inflammatory reaction to circulating pathogens. Although contamination from residual blood cannot be entirely ruled out, the minimal blood content in formalin-fixed, paraffin-embedded tissue makes this an unlikely explanation for the robust mNGS signal observed.

Furthermore, this case underscores the importance of promptly recognizing erythema migrans, which is primarily a clinical diagnosis. Early identification and treatment might have prevented dissemination and hepatic involvement, especially in immunocompromised patients. In this patient, initial IgG serologic testing was borderline positive, but later samples were negative, illustrating the limitations of serologic testing in immunosuppressed patients. Maraspin et al. (*15*) reported that only 28.6% (2 of 7) of rituximab-treated patients with erythema migrans tested positive for *Borrelia* in serologic testing, compared with 62.7%–68.6% in immunocompetent persons. Moreover, *Borrelia* dissemination in those patients was more frequent; isolation rates of *Borrelia* spp. from skin (80%) and blood (40%) were higher than in immunocompetent patients (55%–63% for skin and 2% for blood). Those findings reinforce the need for biopsy-based molecular diagnostics when LB is suspected in immunocompromised patients with negative serology.

In conclusion, this case highlights the importance of considering LB in the differential diagnosis of acute hepatitis, particularly in LB-endemic regions and in patients with epidemiologic risk factors. In immunocompromised patients, negative results of serologic testing do not exclude infection, and invasive diagnostic approaches such as biopsy with molecular testing (PCR, mNGS) might be essential for accurate diagnosis and timely treatment. Noninvasive diagnostic tools such as plasma microbial cell-free DNA sequencing should also be considered.

Appendix Additional information about *Borrelia afzelii* hepatitis in patient treated with venetoclax and obinutuzumab, Switzerland
