# Supplementary material for: Borrelia afzelii Hepatitis in Patient Treated with Venetoclax and Obinutuzumab, Switzerland
Source: Emerg Infect Dis. 2025 Nov;31(11):2167–71. doi: 10.3201/eid3111.250584 (PMC12704518; doi:10.3201/eid3111.250584)
Supplement: Appendix — Additional information about Borrelia afzelii hepatitis in patient treated with venetoclax and obinutuzumab, Switzerland [file 25-0584-Techapp-s1.pdf]

Article DOI: https://doi.org/10.3201/eid3111.250584

EID cannot ensure accessibility for supplementary materials supplied by authors. Readers who have difficulty accessing supplementary content should contact the authors for assistance.

Borrelia afzelii Hepatitis in Patient Treated with Venetoclax and Obinutuzumab, Switzerland

Appendix

Borrelia PCR

A nested polymerase chain reaction (PCR) was conducted using the GeneAmp Fast PCR Master Mix (Thermo Fisher Scientific, 4359187). The reaction used the primers FlaNest1 (5′-TTGGAATGCARCCTGCAA-3′), FlaNest2 (5′-TTGAACAGGTGCAGYCTGA-3′), FlaNest3 (5′-ACACACCAGCRTCACTTTCAGG-3′), and FlaNest4 (5′-CTCCCTCACCAGMRAAMAGATT-3′). PCR amplification was carried out over 35 cycles with an annealing temperature of 64°C. The resulting PCR product was analyzed via capillary electrophoresis using the Qiaxcell Connect system (Qiagen) and subsequently sequenced through Sanger sequencing. The sequence was then analyzed using the NCBI BLAST tool (https://www.ncbi.nlm.nih.gov/) to identify the Borrelia species present in the sample.

Metagenomic sequencing (mNGS) and pathogen detection

A mNGS library were generated using 250 ng of DNA extracted from the liver biopsy sample and the Ion Plus Fragment Library Kit (Thermo Fisher Scientific, 4471252). The resulting library was quantified with the Ion Universal Library Quantification Kit (Thermo Fisher Scientific, A26217) and sequenced on the Ion Torrent GeneStudio S5 platform (Thermo Fisher Scientific). Taxonomic profiling was conducted using CLC Genomics Workbench software (Qiagen), as described by Nienhold et al. (*1*).

References

1. Nienhold R, Mensah N, Frank A, Graber A, Koike J, Schwab N, et al. Unbiased screen for pathogens in human paraffin-embedded tissue samples by whole genome sequencing and metagenomics. Front Cell Infect Microbiol. 2022;12:968135. PubMed https://doi.org/10.3389/fcimb.2022.968135

2. Srivastava B, Gimson A. Hepatic changes in systemic infection. Best Pract Res Clin Gastroenterol. 2013;27:485–95. PubMed https://doi.org/10.1016/j.bpg.2013.06.011

3. Chavanet P, Pillon D, Lancon JP, Waldner-Combernoux A, Maringe E, Portier H. Granulomatous hepatitis associated with Lyme disease. Lancet. 1987;2:623–4. PubMed https://doi.org/10.1016/S0140-6736(87)93009-1

4. Goellner MH, Agger WA, Burgess JH, Duray PH. Hepatitis due to recurrent Lyme disease. Ann Intern Med. 1988;108:707–8. PubMed https://doi.org/10.7326/0003-4819-108-5-707

5. Dadamessi I, Brazier F, Smaïl A, Delcenserie R, Dupas JL, Capron JP. Hepatic disorders related to Lyme disease. Study of two cases and a review of the literature [in French]. Gastroenterol Clin Biol. 2001;25:193–6. PubMed

6. Zanchi AC, Gingold AR, Theise ND, Min AD. Necrotizing granulomatous hepatitis as an unusual manifestation of Lyme disease. Dig Dis Sci. 2007;52:2629–32. PubMed https://doi.org/10.1007/s10620-006-9405-9

7. Middelveen MJ, McClain SA, Bandoski C, Israel JR, Burke J, MacDonald AB, et al. Granulomatous hepatitis associated with chronic *Borrelia burgdorferi* infection: a case report. Research. 2014;1:875. https://doi.org/10.13070/rs.en.1.875

Appendix Table. Review of histological confirmed cases of hepatitis caused by *Borrelia burgdorferi* sensu lato

Article, year of publication	Age	Sex	Country	Comorbidity	Immunosuppression	Manifestation and laboratory tests	Serologic tests for *Borrelia spp.*	Histology	*Borrelia spp*. identified in the liver	Treatment and outcome
Chavanet P et al. (*2*), 1987	46	M	France	No	No	Widespread joint and muscle pain and fever with chills; elevation of liver enzymes (cholestatic parameters >transaminases)	Positive	Granulomatous hepatitis	Not performed	Doxycyclin unclear duration, completely recovered
Goellner MH et al. (*3*), 1988	79	F	USA	None reported	No	Fever, chills, and anorexia; 20 maculopapular erythematous lesions on the abdomen and limbs; elevation of liver enzymes	Positive IgG, negative IgM	Moderate ballooning of hepatocytes, marked hepatocyte mitotic activity, Kupffer cell hyperplasia and sinusoidal infiltration by mononuclear cells and neutrophils; few Dieterle staining spirochetes within hepatic sinusoids and parenchyma	Not performed	Doxycyclin for 3 weeks, completely recovered
Dadamessi I et al. (*4*), 2001	71	M	France	No	No	Fever, icterus, lymphadenopathy cervical and submandibular, polyneuropathy of the lower extremities; elevation of liver enzymes and bilirubin	Positive IgM	Nonspecific, moderate inflammatory sinusoidal and portal infiltration	Not performed	Ceftriaxone for 1 week, completely recovered
Zanchi AC al (*5*), 2007	22	F	USA	None reported	No	Fever, chills, low back and epigastric pain; elevation of liver enzymes (cholestatic parameters >transaminases)	Positive IgM Western blot	Numerous necrotizing granulomas with marked eosinophilic infiltrates; special stains negative for microorganisms	Not performed	Doxycyclin for 8 weeks, completely recovered
Middelveen MJ et al. (*6*), 2014	53	F	USA	Chronic Lyme disease	No	Progressive right upper quadrant pain and fatigue, elevation of liver enzymes (transaminases >cholestatic parameters)	Positive Lyme Western blot	Portal triads with dense mixed inflammatory infiltrate with lymphocytes, plasma cells, occasional eosinophils and prominent non-necrotizing granulomata; Dieterle staining positive for spirochetes and tiny	*Borrelia burgdorferi* sensu strictu	Clarithromycin and Cefdinir

Article, year of publication	Age	Sex	Country	Comorbidity	Immunosuppression	Manifestation and laboratory tests	Serologic tests for *Borrelia spp.*	Histology	*Borrelia spp*. identified in the liver	Treatment and outcome
								vacuolated or granular forms within Kupffer cells.		
Duffau P et al. (*7*), 2022	73	M	France	Marginal zone lymphoma	Bendamustine, Rituximab	Fever; elevation of liver enzymes (cholestatic parameters >transaminases)	Negative	Sinusoidal dilatation (peliosis) and neutrophilic cholangitis/cholangiolitis, Kupffer cell hyperplasia; Warthin Starry stain positive for spirochetes	*Borrelia garinii*	Ceftriaxone for 3 weeks, completely recovered
Our case, 2024	62	F	Switzerland	Chronic lymphocytic leukemia	Obinutuzumab, venetoclax	Multiple erythema migrans, fever, elevation of liver enzymes (cholestatic parameters >transaminases)	Marginally elevated IgG, follow-up negative	Sinusoidal dilatation (peliosis), expanded, markedly edematous portal tracts without fibrosis, and prominent ductular proliferates with neutrophilic infiltrates consistent with neutrophilic cholangitis; special stains negative for microorganisms	*Borrelia afzelii*	Ceftriaxone for 3 weeks, completely recovered
